# Clinical characteristics and treatment modalities in uremic and non uremic calciphylaxis - a dermatological single-center experience

**DOI:** 10.1080/0886022X.2023.2297566

**Published:** 2024-01-04

**Authors:** Sabine Yousuf, Dorothee Busch, Regina Renner, Stefan Schliep, Cornelia Erfurt-Berge

**Affiliations:** a Hautklinik, Uniklinikum Erlangen, Friedrich-Alexander-Universität Erlangen-Nürnberg, Erlangen, Germany; b Private Practice, Esslingen, Germany

**Keywords:** Calciphylaxis, calcific uremic arteriolopathy, nonuremic calciphylaxis, leg ulcer

## Abstract

Calciphylaxis (CP) is a serious, potentially life-threatening disease that presents with medial calcification of small-sized vessels and painful ischemic ulcerations. Although calciphylaxis is frequently seen in patients with end-stage kidney disease on dialysis (calcific uremic arteriolopathy, CUA), there are reported cases of nonuremic calciphylaxis (NUC), which often remain undiagnosed. We conducted a retrospective chart review at our dermatological hospital and evaluated data concerning the epidemiology, comorbidities, medication, laboratory abnormalities, and therapeutic approaches of 60 patients diagnosed with calciphylaxis between 01/2012 and 12/2022. We identified 21 patients diagnosed with NUC and 39 with kidney disease. The predilection sites of skin lesions were the lower legs in 88% (*n* = 53), followed by the thigh and gluteal regions in 7% (*n* = 4). Significant differences were identified in comorbidities, such as atrial fibrillation (*p < 0.001)* and hyperparathyroidism (*p < 0.01)* accounting for CUA patients. Medication with vitamin K antagonists (*p < 0.001)*, phosphate binders (*p < 0.001*), and loop diuretics (*p < 0.01*) was found to be associated with the onset of calciphylaxis. Hyperphosphatemia (*p < 0.001*), increased parathyroid hormone (*p < 0.01*) and triglyceride levels (*p < 0.01)*, hypoalbuminemia (*p < 0.01)* and decreased hemoglobin values (*p < 0.001)* in the CUA cohort were significantly different from those in the NUC group. All patients with CUA received systemic medication. In contrast, only 38% (*n* = 8) of patients with NUC received systemic treatment. Striking discrepancies in the treatment of both cohorts were detected. In particular, NUC remains a disease pattern that is still poorly understood and differs from CUA in several important parameters.

## Introduction

Calciphylaxis is a rare, potentially life-threatening disorder. Owing to its characteristics as an orphan disease (ORPHA:280062), randomized controlled trials on treatment approaches have only been conducted on a small scale, thus providing unsatisfactory data [1,2]. The synonymously used term calcific uremic arteriolopathy describes calciphylaxis-specific necrotic skin ulcers in patients with end-stage chronic kidney disease (ESKD) often treated with dialysis or kidney transplant. However, this expression impedes a holistic approach to all facets of the complex condition, as it fails to elaborate on cases of NUC arising in patients with no evident renal impairment or indications of laboratory abnormalities in bone mineral metabolism [3,4]. Confirmation of typical histological patterns associated with cutaneous calciphylaxis, such as vascular calcification of small-sized vessels, intimal hyperplasia, thrombotic occlusion, epidermal ulceration, and dermal necrosis along with a medical history of nephropathy, can substantiate reasonable suspicion of calciphylaxis. However, Ellis et al. found no significant difference in prevalence of calcification when comparing skin biopsies performed for suspicion of CUA to histological findings in skin biopsies obtained from patients with ESKD without evidence of CUA [5]. Therefore, specificity for defined histological patterns in diagnosis of CUA may be limited. Cases of NUC often represent diagnostic and therapeutic challenges for physicians resulting in misdiagnoses [6–8]. Additionally, treatment options based on pathophysiological findings in patients with CUA are consequently adopted in the form of off-label use and applied to patients presenting with NUC, despite the lack of clear recommendations justifying this approach [9].

## Patients and methods

**Figure 1.**
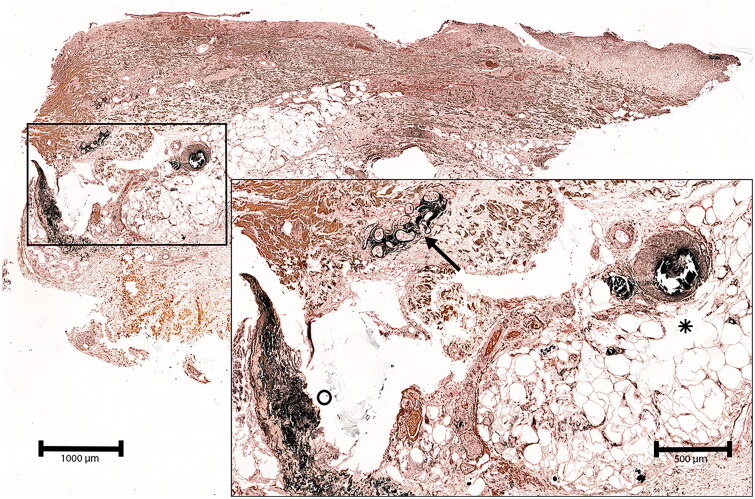
Deep skin biopsy of the left lower leg of a 79 year-old male patient with non-uremic calciphylaxis. The von Kossa stain demonstrates vascular perieccrine calcification (arrow), calcification of a subcutaneous artery and adjacent capillaries (asterisk) as well as diffuse calcification of fibers in the deep dermis.

A retrospective data review was conducted including all patients treated at the Department of Dermatology between 1 January 2012, and31 December 2022, with the ICD-10 diagnosis code E83.50 given initially under the clinically suspected diagnosis of CP. The typical clinical picture consisted of suddenly arising erythematous plaques, which rapidly progressed into extremely painful ischemic ulcers with extensive necrosis, often on the lower leg. In addition, histological confirmation of calciphylaxis, contributing to diagnostic sensitivity, was verified using the von Kossa staining method. The inclusion criteria defined by histopathological hallmarks comprised medial calcification of small-sized vessels, signs of calcification of elastic fibers and eccrine sweat glands, and calcification diffusely distributed in subcutaneous adipose tissue (Figure 1). Initial recruitment involved 140 patients. However, among these patients, 42 were excluded because of deviating clinical or histological findings that were not concordant with the final diagnosis of calciphylaxis during further elaboration and led to differing diagnoses like livedoid vasculopathy or pyoderma gangrenosum. An additional 25 patients were excluded based on histological features that showed histological similarities to cutaneous calciphylaxis but did not meet the criteria for ultimate diagnosis. In the remaining 73 patients, the diagnosis of calciphylaxis was clinically and histologically confirmed. Thirteen patients in the latter group had to be sorted out due to missing basic patient data. Ultimately, 60 datasets of validated calciphylaxis patients were analyzed, evaluated, and further classified into two cohorts consisting of 21 patients diagnosed with NUC and 39 patients diagnosed with CUA. All available data were screened for (a) demographic criteria, such as age, sex, localization, and number of ulcerated skin lesions, (b) comorbidities and medication prior to onset, (c) applied treatment modalities and (d) laboratory abnormalities. This study included data collected solely during regular patient care. The local Ethics Committee approved the study protocol (Approval No. 23-422-Br).

## Statistics

Statistical analyses and evaluations were performed using SPSS version 2.0. With p-values < 0.05 determined to be statistically significant, statistical analysis was performed using the chi-square test for categorical variables and the Student’s *t*-test for quantitative parameters. Laboratory data was acquired on the date closest to the definite diagnosis of calciphylaxis. Descriptive analysis was used to characterize the study population and their treatments.

## Results

In total, we were able to identify and ultimately evaluate 60 patients with a clinical and histological diagnosis of calciphylaxis, treated at our dermatological department over the past 10 years. The characteristics of patients evaluated in this study are shown in Table 1.

**Table 1.** Epidemiological characteristics of all calciphylaxis cases.

Clinical characteristics of CP patients		
	CUA(*n* = 39)	NUC(*n* = 21)
Age(mean)	76 years	70 years
Gender(%)		
Males	49	67
Females	51	33
Location of lesion(%)		
Lower legs	85	95
Thigh	10	0
Lower arm	3	5
Knee	3	0
Affected side of body(%)		
Right	44	43
Left	31	33
Bilateral	26	24
Number of lesions(%)		
Single	74	67
Multiple	26	33
Survival(%)		
Alive	72	95
Dead	28	5
Median Survival(months)		
	60	57

### Demographic data

The 60 patients diagnosed with calciphylaxis were further classified into two groups based on their clinical and histological characteristics (Table 1): a) nonuremic calciphylaxis (35%; *n* = 21) and b) calcific uremic arteriolopathy (65%; *n* = 39). The patients’ ages ranged from 47 to 96 years (median age 74 years) for female and from 50 to 94 years (median age, 70 years) for male patients. A total of 60 CP patients showed a slight male predominance (55%; *n* = 33). No further significant epidemiological differences were observed between the CP and NUC patients.

### Comorbidities

**Figure 2.**
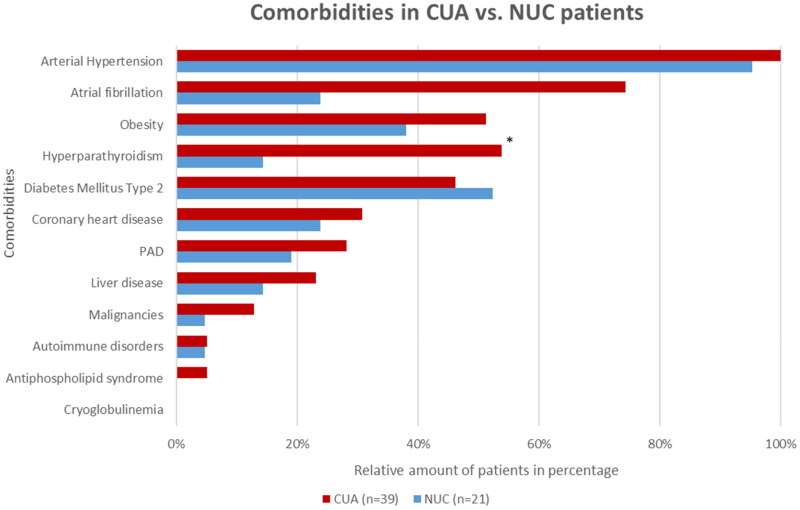
Overview of comorbidities of CUA vs. NUC patients at the time of diagnosis PAD = peripheral artery disease. *Significant (*p < 0.05*) difference between the NUC and CUA cohorts.

Patient charts were reviewed for comorbidities and screened for known risk factors for calciphylaxis according to the current literature (Figure 2). Overall, 65% (*n* = 39) of the patients had concurrent nephropathy, of which only one patient presented with acute kidney failure without signs of chronic renal impairment, whereas the remaining 38 (97%) patients displayed chronic renal insufficiency; with 63% (*n* = 24) patients of them with end-stage kidney disease undergoing dialysis. The majority of patients were treated with hemodialysis (96%; *n* = 23), while only one patient (4%; *n* = 1) received peritoneal dialysis. Of these, 13% (*n* = 5) were known kidney transplant recipients. The most prevalent comorbidities in both groups are shown in Figure 2. Cryoglobulinemia presenting with calciphylaxis-like cutaneous infarcts was ruled out in all patients by the absence of cryoglobulins [10].

Data about smoking habits was not available for all patients. However, a positive smoking history was documented in 6 out of 15 NUC patients (40%) and 11 out of 28 CUA patients (39%).

### Medication

**Figure 3.**
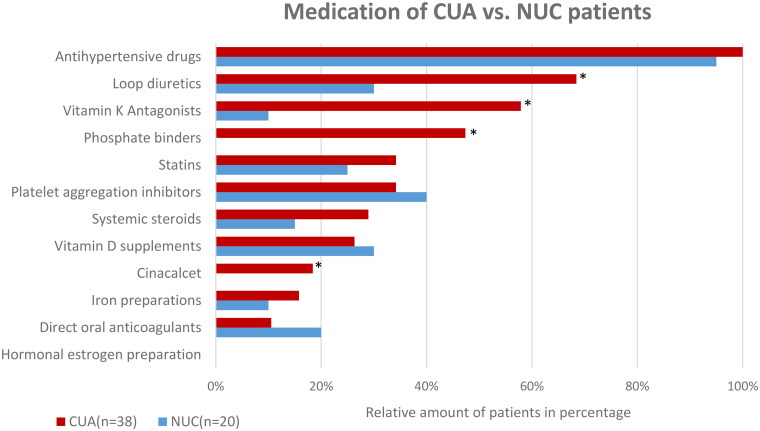
Overview of medication history of CUA vs. NUC patients at the time of diagnosis. *Significant (*p < 0.05*) difference between the NUC and CUA cohorts.

The relative distribution of the prescription medication used by patients in both cohorts is shown in Figure 3. No detailed information about drug history was available for one CUA patient and one NUC patient. They were therefore excluded from further analysis. Accordingly, all CUA (*n* = 38) and 95% (*n* = 20) of NUC patients with a recorded drug history received antihypertensive treatment prior to diagnosis. Interestingly, the second most commonly prescribed drugs by CUA patients were loop diuretics. Distinct differences in overall drug intake were observed in comparison to patients diagnosed with NUC. In contrast to the CUA cohort, a significantly lower intake of vitamin K antagonists was observed in NUC cases (10%; *n* = 2). Furthermore, none of the NUC patients received phosphate binders or cinacalcet prior to disease onset, while seven (18%) CUA patients were treated with cinacalcet, displaying a significantly *(p = 0.04)* higher intake.

### Laboratory findings

As for laboratory parameters, special focus was placed on the values of bone mineral metabolism and renal function, as well as to basic laboratory variables (Table 2). Owing to missing data and the retrospective nature of the study, not all laboratory values could be evaluated for each patient in this study.

**Table 2.** Overview of laboratory abnormalities in CUA vs. NUC patients. Normal ranges for selected parameters: serum calcium 2.2 –2.65 mmol/l, serum phosphate 0.81 – 1.45 mmol/l, calcium-phosphorus-product < 4.40 mmol^2^/l^2^ and serum albumin 35—55 g/l.

	NUC			CUA		
	Number of patients^a^	Laboratory values(%)		Number of patients^a^	Laboratory values(%)	
Laboratory parameters	n	above the normal range	below the normal range	N	above the normal range	below the normal range
Creatinine	n = 21	5	0	n = 39	95	0
eGFR	n = 21	0	5	n = 39	0	95
Calcium	n = 21	5	14	n = 39	3	41
Phosphorus **	n = 17	0	6	n = 38	55	3
Calcium-Phosphorus-Product	n = 15	0	0	n = 36	11	0
Parathyroid hormone**	n = 15	20	7	n = 33	73	3
Albumin**	n = 19	0	21	n = 30	0	57
Glucose	n = 19	42	5	n = 35	49	0
Hemoglobin**	n = 21	5	33	n = 39	0	87
C-reactive protein	n = 21	86	0	n = 38	97	0
Vitamin D	n = 9	0	89	n = 19	0	79
TSH	n = 9	0	0	n = 30	13	0
Iron	n = 9	0	11	n = 25	4	60
Ferritin	n = 8	25	13	n = 24	21	4
INR	n = 19	26	0	n = 39	41	0
HbA1c	n = 17	24	0	n = 27	56	0
Alkaline Phosphatase	n = 20	25	0	n = 37	43	3
AST/ALT	n = 21	24	0	n = 39	0	0
GGT	n = 21	57	0	n = 39	72	0
Triglycerides**	n = 11	0	0	n = 29	31	0
LDL Cholesterol	n = 6	83	0	n = 27	59	0
Cryoglobulins	n = 10	0	0	n = 15	0	0

^a^ Owing to the lack of laboratory data for some cases, the total number of patients for each laboratory parameter may vary.

**Significant (*p < 0.05*) difference between the NUC and CUA cohorts.

## Treatment options

Each patient received stage-appropriate baseline therapy consisting of adequate wound treatment with the application of antiseptics and/or antimicrobial wound dressings, surgical debridement when necessary, and sufficient pain management. Antibiotics were administered if signs of wound infection were present. Whenever possible, vitamin K antagonists were discontinued and switched to direct oral anticoagulants, including factor Xa inhibitors, because a significant and critical association with the development of the condition has been described [11,12]. Unfortunately, for some patients suffering from autoimmune disorders, such as antiphospholipid syndrome, intake of Vitamin K antagonists cannot be discontinued after careful risk assessment. Systemic treatments for our cohort are shown in Table 3. All CUA patients (*n* = 39) were given multimodal and interdisciplinary systemic treatment in cooperation with our in-house Nephrological Department. In contrast, only 38% (*n* = 8) of NUC patients received systemic therapies. Particularly noteworthy is the initiation of treatment with acetylsalicylic acid at a dose of 300 mg daily in selected NUC patients (29%; *n* = 6) based on an observation in one NUC case in which a higher dose of acetylsalicylic acid was administered during a cardiac intervention, leading to a subsequent improvement of the NUC wounds. Although this off-label use does not comply with any commonly applied therapeutic measures, preliminary results are promising. Amongst the aforementioned 6 patients, 67% (*n* = 4) experienced substantial improvements in skin lesion regression, while complete remission of skin ulceration was achieved in the remaining 33% (*n* = 2) of patients. Interestingly, a higher proportion of patients with NUC underwent surgical debridement (14%; *n* = 3) than those with CUA (8%; *n* = 3).

**Table 3.** Systemic treatment in patients with NUC and CUA.

	Number of patients treated *	
Treatment	NUC	CUA
Any systemic therapy	8 (38 %)	39 (100 %)
Sodium thiosulfate	n.a.	14 (38 %)
Cinacalcet	n.a.	11 (28 %)
Sevelamer carbonate (phosphate binder)	n.a.	6 (15 %)
Lanthanum carbonate (phosphate binder)	n.a.	5 (13 %)
Biphosphonates	2 (10 %)	2 (5 %)
Vitamin K supplementation	n.a.	3 (8%)
Acetylsalicylic acid^a^ (as a therapeutic intervention)	6 (29 %)	n.a.

^a^ some patients received more than one systemic treatment during course of disease.

** for rationale of this therapeutic invention, see results section.

n.a. - not applied in these patients.

## Discussion

In recent literature, data on information about epidemiology, comorbidities, and treatment options for NUC cases is scarce. In contrast to larger nephrological studies [13] the proportion of NUC patients among all CP patients in our study is higher than the proportion reported in the literature. This may be due to the dermatological focus of this work.

### Epidemiology

Although our study did not comply with previous data identifying female sex as a risk factor for calciphylaxis [14–16], the median age of patients being above 50 years aligned with the findings of other case studies [3,17]. Consistent with previous reports by Fernandez et al. [18], the vast majority of our patients presented with typical skin lesions on the lower legs. Furthermore, we primarily observed single lesions, although cases of calciphylaxis presenting with multiple lesions have been reported [14]. Our case study revealed a mortality rate of 83% at 12 months, of which 92% suffered from CUA, while a single patient belonged to the NUC cohort. This confirms the literature about high mortality rates associated with calciphylaxis, particularly within the first year after onset of the disease [3,15].

### Comorbidities and risk factors

#### Metabolic disorders

Conditions of metabolic syndrome, such as diabetes mellitus [14,16,19], obesity [3,16,20], and arterial hypertension [19], as well as cardiovascular conditions related to metabolic disorders, such as coronary artery disease or atrial fibrillation treated with vitamin K antagonists [19], have been noted as significant risk factors contributing to the development of CUA. Our findings regarding the most prevalent comorbidities in the CUA cohort were consistent with those previous reports. In patients with NUC, Nigwekar et al. identified primary hyperparathyroidism, malignancies, and alcoholic liver disease as the most prevalent comorbidities in a review of 36 NUC cases [7]. Our cohort of NUC patients did not support these findings, as the most predominant comorbidities were arterial hypertension (95%), diabetes mellitus (52%), and obesity (44%). Regarding NUC with concomitant arterial hypertension, the entity ‘Martorell hypertensive ischemic leg ulcer’ must be discussed as an important differential diagnosis, since both conditions display similar clinical and histological patterns, suggesting a common origin [21]. Hafner et al. pragmatically suggest the division of this type of ulceration in patients without ESKD into two groups with proximal and distal patterns [21]. A proximal manifestation of the ulceration is assumed to be NUC, whereas distal involvement is assumed to be a hypertensive ischemic leg ulcer. For the cases included in our analysis, we intensively discussed the diagnosis within the authors’ team and made an NUC diagnosis based on the clinical and histopathological images. Quantifiable diagnostic criteria for distinguishing between NUC and hypertensive ischemic leg ulcers are lacking. Lastly, malignancies represented the least frequently determined comorbidity in our NUC cohort, as only confirmed in a single patient, which contradicts the findings of Nigwekar et al. [7]. In contrast, a single case review conducted by Gomes et al. revealed diabetes mellitus and obesity to be possible triggers of NUC, as frequently confirmed in our patients, although not proven to be statistically significant [22].

#### Disorders in bone and mineral metabolism

There is strong evidence of abnormalities within the bone mineral disease axis in patients with CUA, also known as chronic kidney disease-mineral and bone disorder (CKD-MBD). These patients are mainly treated with dialysis. Laboratory abnormalities significantly raising the risk for the development of calciphylaxis include hyperparathyroidism [23,24], hypercalcemia [17,23], hyperphosphatemia [14,15,20,23], an elevated calcium-phosphorus product [3], and hypoalbuminemia [15,17,20,25]. Analysis of our laboratory data confirmed statistically significant deviations regarding hyperparathyroidism, hyperphosphatemia, and hypoalbuminemia in patients with CKD-MBD, consistent with the results of previous studies. However, regarding serum calcium levels, we observed contradictory results, since hypocalcemia was diagnosed in 41% of our CUA cohort, and only a single patient showed hypercalcemia in laboratory analysis. Similarly, no statistically significant elevated calcium-phosphorus product levels were identified in our cohort, corresponding to the findings of Hayashi et al. [17] and Brandenburg et al. [1]. The rationale as to why normal calcium-phosphorus-product levels are described in many cases is due to severe calcification inhibitor deficiencies, which cause calcium and phosphate to rapidly deposit in tissues. Consequently, elevations in serum calcium, serum phosphate, and calcium-phosphorus product concentrations are no longer detectable, which implies that these laboratory parameters may be misleading indicators to assess the risk of calciphylaxis, since parameters of bone and mineral disorders may be alternating [1,26]. Further, Santos et al. analyzed information from an internet-based registry about 117 CUA patients and found rather modest deviations of parameters of bone and mineral metabolism at the time of diagnosis [27]. The lack of hypercalcemia in NUC patients has also been described in other case series [11] and only proves relevant in NUC cases associated with hyperparathyroidism. Although not significant, hypoalbuminemia was found in 21% of our patients as the most striking parameter, while none showed an increased calcium-phosphate product or other significant laboratory deviations. Hypoalbuminemia has been identified as a risk factor in CUA [28] and has also been reported in other NUC cases [7] in accordance with our observation, yet no explanation for this phenomenon is known. Zhang et al. [29] raised the question of whether albumin-corrected serum calcium needs to be assessed more closely in order to evaluate calcium levels in CUA patients with regard to their significance as a risk factor [30]. Our findings confirm that laboratory values for bone mineral metabolism in cases of NUC often do not present any abnormalities and are highly variable [31].

### Medication

#### Vitamin K antagonists

Previous data explicitly focused on the effects of certain medical drugs on the development of calciphylaxis and extensively classified vitamin K antagonists as high-risk medications [17,19,32,33]. Holden et al. [34] showed that coumarin-derived anticoagulants promote vascular calcification processes in patients treated with hemodialysis by inhibition of vitamin K-dependent Matrix Gla protein, which is crucial to avoid arterial calcium deposition [35]. In our cohort of CUA patients, 58% were treated with vitamin K antagonists. In contrast, in the NUC cohort, only 10% received therapy with vitamin K antagonists, which is inconsistent with data indicating a link between NUC and vitamin K antagonists [12]. A subgroup analysis of 19 patients with NUC revealed no significant association between the intake of vitamin K antagonists and the development of NUC [36]. It is therefore hypothesized that CUA-associated high-risk medications may not entirely correlate with NUC. As therapy with vitamin K antagonists also increases the risk of stroke in hemodialyzed patients [37], Benett et al. investigated whether dialyzed patients should receive vitamin K antagonists and concluded that prescriptions warrant strict indications [38]. Accordingly, Eiser et al. suggested an individual risk stratification including assessment of the CHA_2_DS_2_-VASc-scoring system, antiplatelet therapy in patients not receiving oral anticoagulants, and evaluation of known risk factors of calciphylaxis as a precondition before therapy with coumarin derivatives is initiated [33]. Although scientific research is currently limited, a retrospective analysis identified the factor Xa inhibitor apixaban as a safe and effective alternative for patients with ESKD on dialysis and calciphylaxis [39].

#### Systemic corticosteroids and loop diuretics

Systemic corticosteroid therapy represents another reported risk factor that favors the development of calciphylaxis [3]. In our analysis, 29% of patients with CUA and 15% of patients with NUC were treated systemically with corticosteroids at the time of diagnosis, although the difference was not statistically significant. More strikingly, a significant difference was identified concerning loop diuretics, with 68% of CUA patients and 30% of NUC patients treated with this group of drugs during the development of calciphylaxis. Since there is no evident scientific data elucidating on the correlation between loop diuretics and calciphylaxis to our knowledge, this group of medical drugs should be noted for further investigations as a possible trigger of calciphylaxis but might be an outlier observation in this study.

### Therapeutical approaches

A number of therapeutic strategies and several off-label treatments has been initiated in a few patient series, with varying results [40–47]. The high frequency of debridement as a therapeutic approach for NUC shown in our study can be explained by the fact that patients with NUC are generally undertreated with systemic therapies, resulting from missing therapeutic recommendations. However, some authors fear that excessive manipulation of the ulcerated lesions may cause a kind of pathergy phenomenon analogous to pyoderma gangrenosum, and therefore also question the collection of a tissue sample [48]. A monocentric retrospective study of 64 patients showed a significantly higher 1-year survival rate when radical surgical debridement was performed compared with the group of patients without this debridement measure [3]. Due to the retrospective nature of this study, detailed data on the outcomes of specific therapies were not available in this study setting.

#### Sodium thiosulfate

Sodium thiosulfate (STS) has emerged as a possible cornerstone in calciphylaxis therapy, specifically CUA. Promising results regarding treatment effectiveness and efficacy have been shown [40–42]. However, a meta-analysis could not show an effect of STS on skin lesion improvement or survival benefit in CUA [49]. Different application schemes are recommended [50]. In our data analysis, most patients with CUA were treated with sodium thiosulfate infusions. STS is hypothesized to have chelating and antioxidant properties, thus leading to the chelation of calcium to form calcium thiosulfate in patients with CP. Since calcium deposits are proven to have increased solubility compared to other calcium salts, they are more readily removed from the body, thereby promoting the disposal of excess calcium from the afflicted vessel [51,52]. Although no patient with NUC in our cohort was treated with STS infusions, a case report by Generali et al. reported complete resolution of the skin lesion after therapy in a NUC patient treated with STS [53]. When initiating treatment with STS, patients should be evaluated for adverse reactions such as metabolic acidosis [54,55] and although it is commonly used, its efficacy remains to be proven by further studies (i.e., Clinical Trial NCT03150420).

#### Other therapeutic approaches

In recent years, various other therapeutic regimen have been used to treat calciphylaxis. Several case reports have confirmed that intravenous administration of bisphosphonates, specifically pamidronate infusions, is an effective treatment option in patients with ESKD [56,57]. A positive effect of pamidronate has also been reported in patients with NUC [58]. Data regarding other successful therapeutic strategies in the treatment of CUA and occasionally applied in our CUA cohort include treatment with cinacalcet as a single or combination therapy [59], oral and intravenous treatment with vitamin K1(45) and treatment with phosphate binders such as lanthanum carbonate [60]. Furthermore, a therapeutic approach with topical, intramuscular and intravenous application of amnion-derived mesenchymal stem cells in treatment of a patient with CUA showed a promising outcome [61]. These results may spark hope for the successful treatment of CUA patients, yet there are hardly any scientific sources validating these approaches for the treatment of NUC.

## Limitations

This study was limited by its retrospective design. The documentation of medical history was insufficient for some patients, leading to a lack of data in certain cases. Due to missing follow-up visits and poor prognosis of the condition, treatment efficacy could not be analyzed in the treated patients.

## Conclusion

In conclusion, our retrospective study confirms that patients with NUC differ from CUA patients regarding epidemiology, comorbidity, and concomitant medication. Current diagnostic criteria are insufficient to clearly distinguish NUC from other entities, such as hypertensive ischemic leg ulcers or vasculopathic ulcers. Based on our results, further research on the identification of pivotal risk factors for both variants of calciphylaxis is vital. Further single and combination therapies must be assessed and elaborated in regard to efficacy and safety, as well as for principles of dosage, treatment duration, adverse effects, and outcomes by randomized controlled studies.

## Data Availability

The abstract contains 237 words, and the major text of the article contains 3261 words, three tables, three figures, and 61 references.
